# Engineering electrospun multicomponent polyurethane scaffolding platform comprising grapeseed oil and honey/propolis for bone tissue regeneration

**DOI:** 10.1371/journal.pone.0205699

**Published:** 2018-10-29

**Authors:** Cui Yan Chao, Mohan Prasath Mani, Saravana Kumar Jaganathan

**Affiliations:** 1 Department of Rehabilitation Medicine, The First Affiliated Hospital of Xi’an Jiao Tong University, Xi’an, Shaanxi, PRC China; 2 School of Biomedical Engineering and Health Sciences, Faculty of Engineering, Universiti Teknologi Malaysia, Skudai, Malaysia; 3 Department for Management of Science and Technology Development, Ton Duc Thang University, Ho Chi Minh City, Vietnam; 4 Faculty of Applied Sciences, Ton Duc Thang University, Ho Chi Minh City, Vietnam; 5 IJNUTM Cardiovascular Engineering center, School of Biomedical Engineering and Health Sciences, Faculty of Engineering, Universiti Teknologi Malaysia, Skudai, Malaysia; Institute of Materials Science, GERMANY

## Abstract

Essential oils play an important role in reducing the pain and inflammation caused by bone fracture.In this study, a scaffold was electrospun based on polyurethane (PU), grape seed oil, honey and propolis for bone tissue-engineering applications. The fiber diameter of the electrospun PU/grape seed oil scaffold and PU/grape seed oil/honey/propolis scaffold were observed to be reduced compared to the pristine PU control. FTIR analysis revealed the existence of grape seed oil, honey and propolis in PU identified by CH band peak shift and also hydrogen bond formation. The contact angle of PU/grape seed oil scaffold was found to increase owing to hydrophobic nature and the contact angle for the PU/grape seed/honey oil/propolis scaffold were decreased because of hydrophilic nature. Further, the prepared PU/grape seed oil and PU/grape seed oil/honey/propolis scaffold showed enhanced thermal stability and reduction in surface roughness than the control as revealed in thermogravimetric analysis (TGA) and atomic force microscopy (AFM) analysis. Further, the developed nanocomposite scaffold displayed delayed blood clotting time than the pristine PU in the activated prothrombin time (APTT) and partial thromboplastin time (PT) assay. The hemolytic assay and cytocompatibility studies revealed that the electrospun PU/grape seed oil and PU/grape seed oil/honey/propolis scaffold possess non-toxic behaviour to red blood cells (RBC) and human fibroblast cells (HDF) cells indicating better blood compatibility and cell viability rates. Hence, the newly developed electrospun nanofibrous composite scaffold with desirable characteristics might be used as an alternative candidate for bone tissue engineering applications.

## Introduction

In clinical applications, every year the bone tissue transplant surgery was increased in number insisting the demand for bone tissue grafts. The commercial bone tissue grafts used in the reconstruction of the bone defects were autografts and allografts. But their application in the biomedical applications were reduced owing to many limitations such as increased surgery time, donor site pain, and limited quantity of harvestable bone [1–4]. Hence, it forces the researchers to search for the alternate material (especially artificial tissue scaffolds) as a substitute for bone repair. The scaffold for bone tissue engineering must be biocompatible, porous, enough mechanical strength and proper degradation rate. Further, the developed scaffold must mimic the ECM, to support the tissue structure and also cells to adhere and migrate [5, 6].

In bone tissue engineering, a wide range of polymers were used to fabricate the bone scaffolds. Among, wide range of polymers, the polyurethane (PU) is used in this study to develop a scaffold for bone tissue engineering. PU is a copolymer which composed of both soft and hard segments. It was widely used in tissue engineering applications owing to its biodegradability, good barrier properties and better oxidation stability [7, 8]. Further, by tuning soft and hard segments, the properties of the PU were easily changed and been utilized for various biomedical applications. In general, there are different techniques utilized for the fabrication of PU tissue scaffolds such as electrospinning, solvent casting, freeze drying, batch foaming and injection foaming. In this research, the electrospinning technique was utilized for fabricating the PU scaffolds [1].

Electrospinning is a versatile and cost effective technique which utilizes the high voltage to produce the produce nonwoven nanofibers. The nonwoven nanofibers were found to have high-surface area-to-volume ratio and high porosity which can mimic the native structure of the extracellular matrix of bone tissue [9]. In this technique, by applying high voltage to the syringe needle, the fine nanofibers will draw from the polymer solution and collected on the aluminum foil which is placed on the collector drum [10]. Electrospinning involves many parameters such as applied voltage, flow rate, viscosity of polymer solution and collector distance which influence the fiber diameter of the nanofibers [11, 12].

In bone tissue engineering various types of innovations have been made to support the bone tissue repair. Miculescu et al presented the starch particles incorporated hydroxyapatite scaffolds for medical applications. The reported that the starch incorporated hydroxyapatite composite was considered to be safe in terms of toxicity and might utilized for bone cements, bone waxes, adhesives or scaffolds [13]. Some studies have reported the several metallic nanoparticles such as ZnO, SiO_2_ incorporated composites for biomedical and high performance applications. The addition of nanoparticles showed the improved properties, excellent antibacterial activity, improved cell adhesion and osteoconductivity properties [14–16]. In another study, Voicu et al prepared cellulose acetate membrane for biomedical applications added with sericin. The fabricate membranes showed good osteoblast cell response suggested it as a suitable candidate for osseointegration processes [17]. Hence, these type of works motivated us to study the effect of grapeseed oil, honey and propolis in the bone tissue engineering. The pain and inflammation is created at the tissue near the fracture site when the bone get fractured. They are many treatments available to reduce the pain and inflammation occurred at the fracture site. In early days, the essential oil is used to get relief from the pain at the fracture site. Hence, in this research the grapeseed oil was used to fabricate the bone scaffold. Grape seed oil is obtained from the seeds of grapes and it was a by-product of wine making [18]. It was widely used as cooking oil and also in skin care applications as a cosmetics. Grape seed oil contains 0.8 to 1.5% of phenols and steroids and small amounts of vitamin E [19, 20]. The grape seed oil was reported to possess highest antioxidant capacity (42.18 mmol of Trolox equivalent/g) which was due to the existence of high amount of gallic acid, epicatechin, catechin, proanthocyanidins and procyanidins [21, 22]. The polyphenols present in grape seed oil have ability to inhibit the inflammatory response by preventing the release of arachidonic acid (AA) [21, 23]. Further, the phenolic component present in the grape seed oil was found to be toxic on both bacteria’s (*Staphylococcus aureus* and *Escherichia coli*) concluding its antimicrobial effect [21]. In addition to grape seed oil, the propolis is added with grape seed oil to improve the bioactivity in the PU polymer. Further, in a recent study, it was that the observed that the incorporation of propolis and honey into the electrospun membrane resulted in the hydrophilicity behavior [24]. Propolis is a resinous substance obtained by bees from their salivary secretions [25]. The propolis were reported to have many bioactive constituents such as flavonoid, phenolic components, amino acids and some inorganic compounds [26]. Further, it possess various medicinal properties like high adhesive, antibacterial, antifungal, antiviral, antioxidant and anti-inflammatory activities [25]. Honey is produced by bees and it contains numerous biological components like glucose, fructose, sucrose, water, amino acids, vitamins, minerals, and enzymes [27]. Honey on a whole or its constituents have been widely reported to possess various biological and pharmacological properties from wound healing to anti-tumour and from anti-inflammatory to antibacterial activities [28–30]. Further, the components such as flavonoids and phenolic acids present in the honey are reported to have synergistic antioxidant effect [31, 32]. In a recently concluded clinical trial, honey was tested on 102 patients with chronic wounds and ulcers which was not able to cure using conventional wound healing treatment. The results of this study showed that honey could heal these wounds dramatically in 4–7 weeks completely insinuating its wound healing potential [33]. Similarly, a recent study also showed the ability of honey to heal the mandibular bone defects of the Wistar rats efficiently compared to untreated control group [34]. In this study, PU nanofiber containing grape seed oil, honey and propolis were electrospun using electrospinning technique. For the first time, the combination effect of hybrid scaffolds based on honey and propolis with the grape seed oil was studied. For the fabricated PU and PU/grape seed oil/honey/propolis scaffold, the physiochemical characteristics, blood compatibility parameters and cytocompatibility studies were investigated to analyze its effect for bone tissue engineering.

## Materials and methodology

### Materials

The medical grade Tecoflex EG-80A polyurethane was obtained from Lubrizol and dissolved in dimethylformamide (DMF) solvent (Sigma Aldrich, UK). The grape seed oil, honey and propolis were obtained locally. The chemical phosphate buffered saline (PBS) and sodium chloride physiological saline (0.9% w/v) were purchased from Sigma-Aldrich, Kuala Lumpur, Malaysia. The reagents such as rabbit brain activated cephaloplastin, calcium chloride (0.025 M), and thromboplastin (Factor III) used in the blood compatibility studies were purchased from Diagnostic Enterprises, Solan, India.

### PU and PU composite solutions

For fabricating PU membrane, 9 wt% of PU solution was prepared by dissolving calculated amount of PU in DMF and stirred overnight at room temperature to obtain clear homogeneous solution. Similarly, the grape seed oil, honey and propolis homogeneous solution were prepared for 9 wt% obtained by adding calculated amount of grape seed oil, honey and propolis in DMF and stirred 1 hr maximum. For fabricating PU/grape seed oil solution, the prepared 9 wt% of PU solution was doped with 9 wt% of prepared grape seed oil solution in the ratio of 7:2 (v/v%). Similarly, for PU/grape seed oil/honey/propolis solution, the prepared 9 wt% homogeneous PU solution was mixed with 9 wt% of homogeneous grape seed oil, honey and propolis solution at a ratio of 7:1:1 (v/v%) respectively. In composite solution preparation, the solutions were stirred for 2 hrs maximum at room temperature for even dissolution.

### Electrospinning technique

The prepared solutions were placed in 10 ml syringes with 18-G stainless steel needle and loaded in the syringe pump. All the prepared PU and composite solutions were electrospun at a voltage of 10.5 kV with flow rate of 0.5 ml/h and collector distance placed at 20 cm. The nanofibers were obtained on the aluminum foil were carefully detached and dried under vacuum at room temperature to remove any residual DMF content.

### Physio-chemical characterizations

#### Scanning electron microscopy (SEM)

SEM analysis was performed to observe the morphological details of electrospun membranes. Prior to capturing photomicrographs, the samples were gold coated. Then, the coated samples were imaged to obtain the SEM images of the electrospun membrane and the average fiber diameter was calculated using Image J software.

#### Contact angle measurements

The water contact angle measurements were measured using VCA Optima contact angle measurement unit to determine the wetting ability of the electrospun scaffold. To begin, a small piece of electrospun membrane was paced on the measuring surface and water droplets of 0.5 μL were dispensed on the testing membrane and the static image of the water droplet on the testing membrane was captured high-resolution video camera. The contact angle was measured automatically using computer integrated software.

#### Mechanical testing

The mechanical properties of fabricated scaffolds were tested using uniaxial testing machine. To begin, the rectangular samples with size 40 mm× 15 mm were cut and mounted between the grips. After gripping, the deformation was measured at a cross head speed of 5 mm min^−1^ with load of 500 N. Finally, the tensile strength and elongation at break were determined from the resulting stress/strain curves.

#### Fourier transform infrared spectroscopy (FTIR)

The chemical compositions of the prepared composites were inspected using a FTIR analysis. A small piece of electrospun membrane was placed on the sensor surface and the spectra was inspected. The spectra was recorded at wavelength of 600–4000 cm^−1^ at 4 cm^−1^ resolution with average of 32 scans per minute. The spectra was baseline corrected and the peaks were identified using Speckwin software.

#### TGA analysis

The thermal stability of the electrospun membrane was studied using PerkinElmer TGA 4000 unit. A small piece of sample (3 mg) was placed on the measuring unit and the heating rate was performed. The TGA analysis was carried out under dry nitrogen atmosphere at an ascending rate of 10°C/min with temperature range of 30–1000°C. TGA and DTG curve was drawn from the obtained data’s using excel sheet.

#### AFM analysis

To calculate the surface roughness, AFM analysis was performed using Nanowizard, JPK instruments. A small piece of sample was placed on the scanning surface and the scanning was performed at room temperature in normal atmosphere. The scanning was performed in 20 * 20 μm sizes the 3D image with 256 * 256 pixels was captured using JPKSPM data processing software.

### Blood compatibility analysis

#### APTT and PT assay

APTT assay was used to determine the intrinsic pathway of the blood clot while the PT assay determines the extrinsic pathway. To begin the APTT assay, the developed samples were washed with PBS and incubated at 37°C for 30 min. After incubation, the samples were mixed with 50 μl of PPP for 1 min 37°C followed by adding 50 μL of rabbit brain cephaloplastin reagent and CaCl_2_ (0.025 M) solution for 3 min 37°C. The mixture was gently stirred which results the formation of the blood clot and APTT was measured. For the PT assay, the procedures were same as the APTT assay where the electrospun membrane mixed with 50 μl of PPP was further incubated with 50 μl of thromboplastin reagent (Factor III) for 3 min at 37°C. Finally, the blood clot formation was done by stirring and PT was measured [35].

#### Hemolysis assay

To start the assay, the electrospun membrane (1 cm ×1 cm) was soaked in 0.9% w/v of saline at 37°C for 30 min. After soaking, the samples added with the mixture of citrated blood and diluted saline (4:5 v/v%) for 1 h at 37° C. Then, the samples centrifuged at 3000 rpm for 15 min and OD was measured. The absorbance was measured at 542 nm with pipetted supernatant which indicates the release of hemoglobin. The percentage of hemolysis or hemolytic index was calculated as described earlier [35].

### Cell culture and MTS assay

The cell viability of the electrospun scaffolds were determined using HDF cells. Initially, HDF were cultured using DMEM medium with 10% fetal bovine serum and incubated at 37°C and 5% carbon dioxide (CO_2_). The medium was replaced for every 3 days. To begin the cell seeding onto the electrospun scaffold, the samples were cut and placed in the 24 well plates and sterilized with 75% of alcohol solution. After sterilizing, the scaffolds were washed with PBS solution. Then, HDF cells with density of 1.0×10^5^ cells were seeded per scaffold in each well and incubated at 37°C with 5% CO_2_. The MTS assay was used to determine the cell viability rates in the electrospun membranes after 72 hr incubation. After 3 days culture, the cell seeded scaffolds were washed with PBS and added with 20% of MTS solution (3-(4,5-dimethylthiazol-2-yl)-5-(3-carboxymethoxyphenyl)-2-(4-sulfophenyl)-2Htetrazolium, inner salt) and further incubated for 4 hr. After 4 hr, the culture plates were retrieved and absorbance was measured at 490 nm using spectrophotometer to measure the cell counts in the fabricated membranes.

### Statistical analysis

All experiments were performed thrice independently and the Unpaired t-test was carried out to calculate the statistical significance. All experiment results are expressed as mean ± SD and for qualitative experiments, a representative of three images is shown.

## Results

### SEM investigation

**Fig 1:**
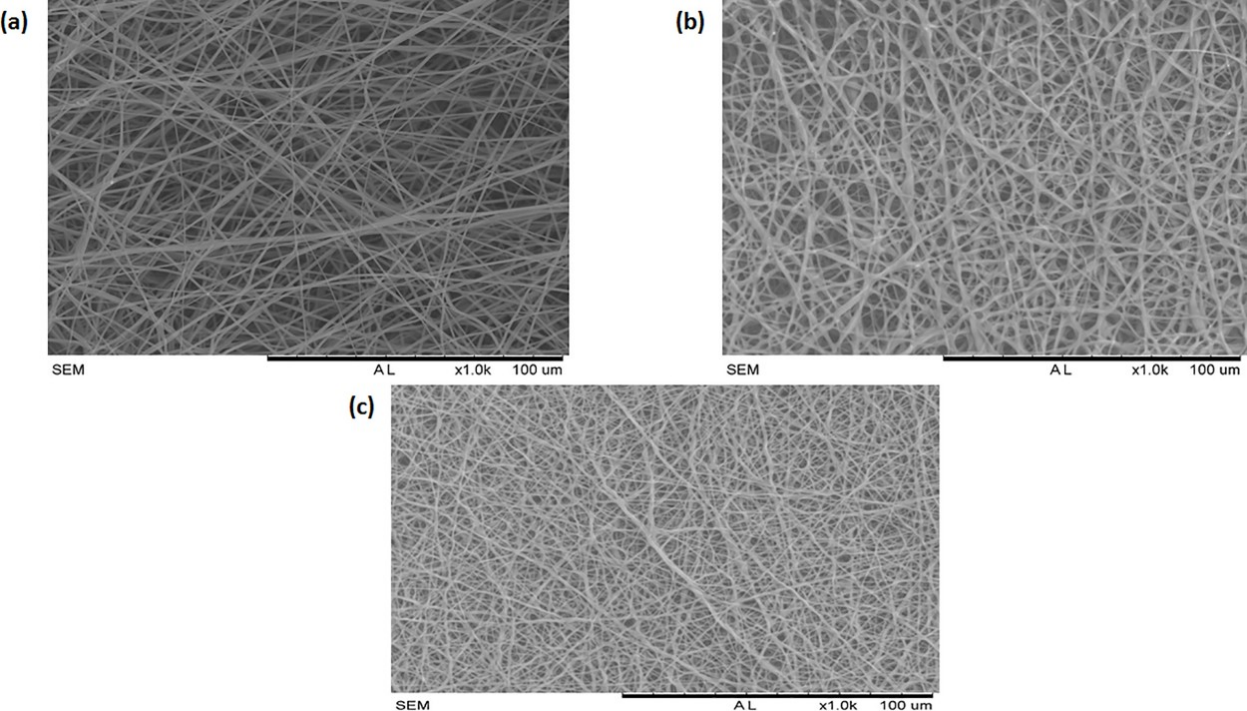
SEM images of a) Polyurethane b) Polyurethane/ grape seed oil composites c) Polyurethane/grape seed oil/propolis/honey composites.

**Fig 2:**
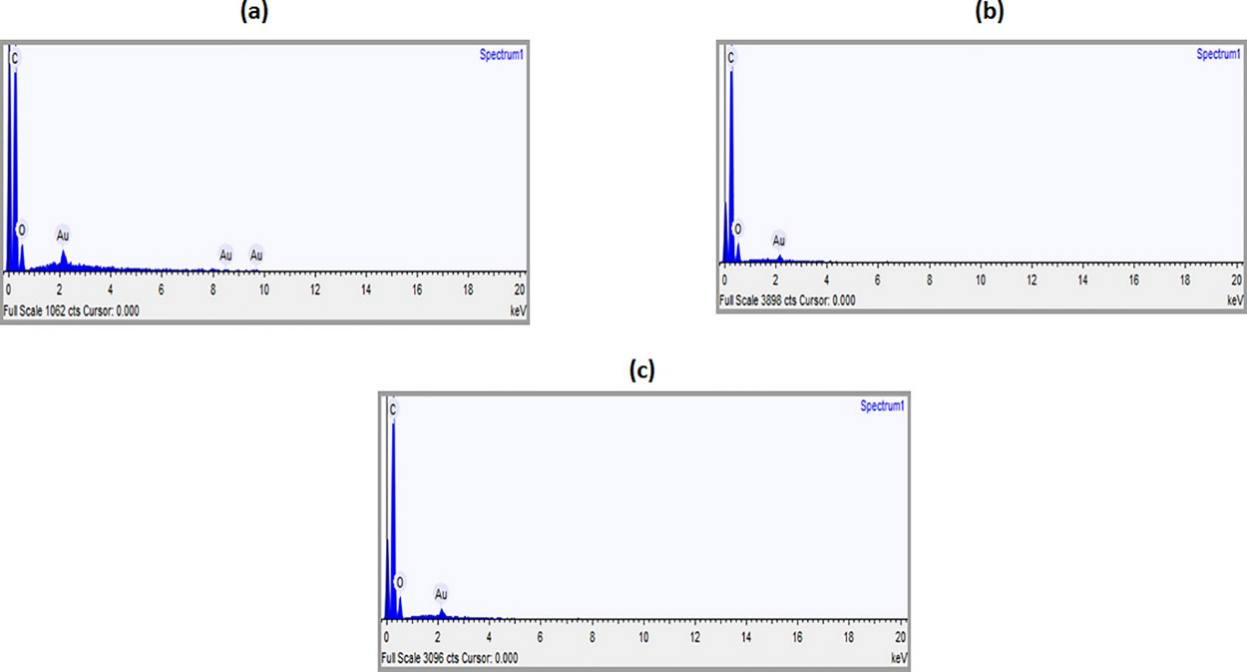
EDS spectrum of a) Polyurethane b) Polyurethane/ Grape seed oil composites c) Polyurethane/Grape seed oil/propolis/honey.

**Fig 3:**
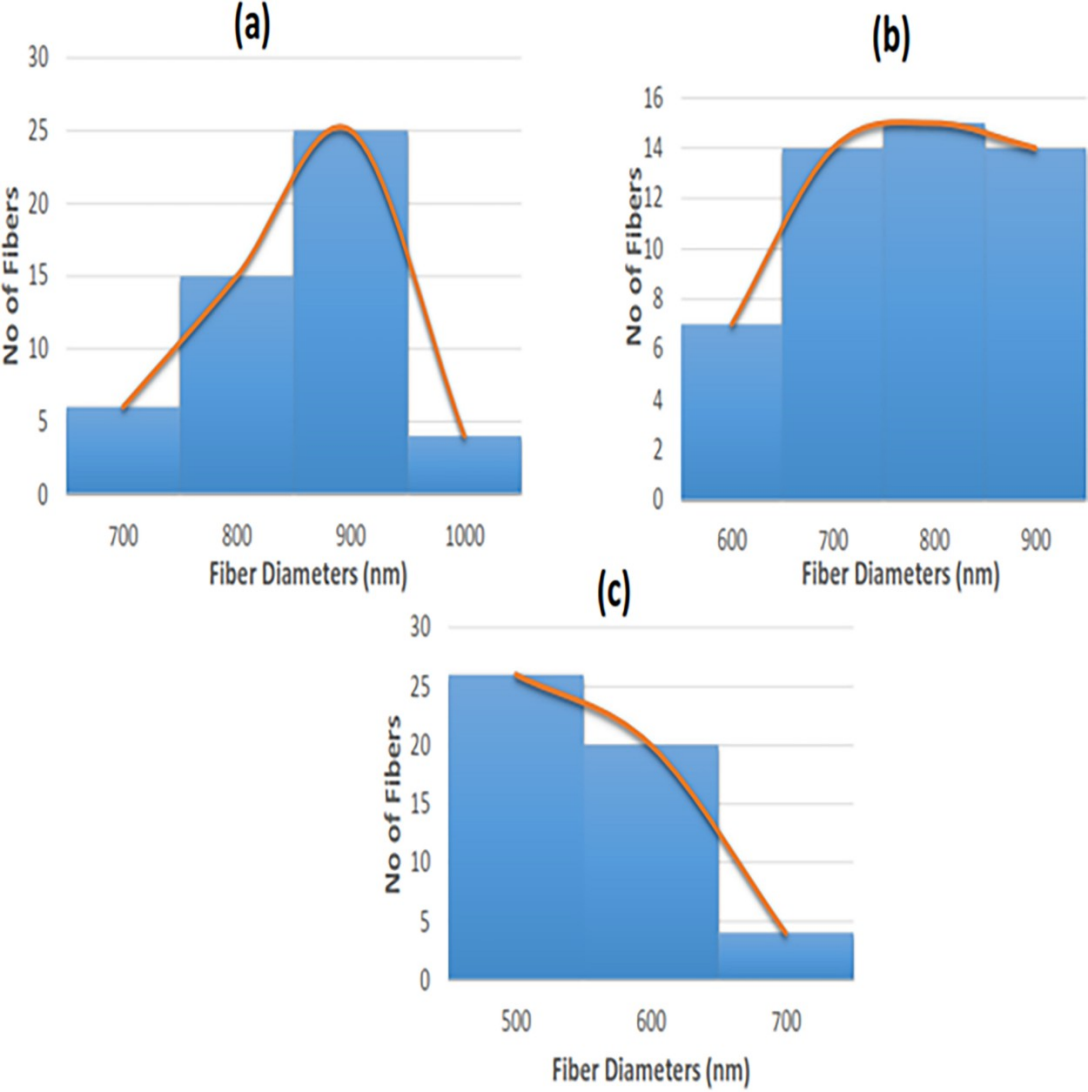
Fiber diameter distribution of a) Polyurethane b) Polyurethane/ grape seed oil composites c) Polyurethane/grape seed oil/propolis/honey composites.

The morphology and EDS of the electrospun PU, PU/grapeseed oil and PU/grape seed oil/honey/propolis scaffold were shown in Figs 1 and 2. Further, Tables 1–3 depicts the elemental analysis for the electrospun PU, PU/grapeseed oil and PU/grape seed oil/honey/propolis scaffold. From the SEM image, it was observed that the prepared PU, PU/grape seed oil and PU/grape seed oil/honey/propolis scaffolds showed uniform fibers without any beads. The fiber diameters of electrospun PU, PU/grape seed oil and PU/grape seed oil/honey/propolis scaffold were found to be 890 ± 116.911 nm, 817 ± 155.45 nm and 601 ± 151.76 nm respectively and the distribution curve was shown in Fig 3.

**Table 1:** Elemental analysis of PU membrane.

Element	Weight (%)	Weight (% σ)	Atomic (%)
**Carbon**	70.717	1.290	79.645
**Oxygen**	23.614	1.237	19.966

**Table 2:** Elemental analysis of PU/Grapeseed oil composite.

Element	Weight (%)	Weight (% σ)	Atomic (%)
**Carbon**	77.212	0.745	83.349
**Oxygen**	20.348	0.728	16.490

**Table 3:** Elemental analysis of PU/Grapeseed oil/Propolis/Honey composite.

Element	Weight (%)	Weight (% σ)	Atomic (%)
**Carbon**	73.942	0.771	81.057
**Oxygen**	22.749	0.754	18.722

### FTIR analysis

**Fig 4:**
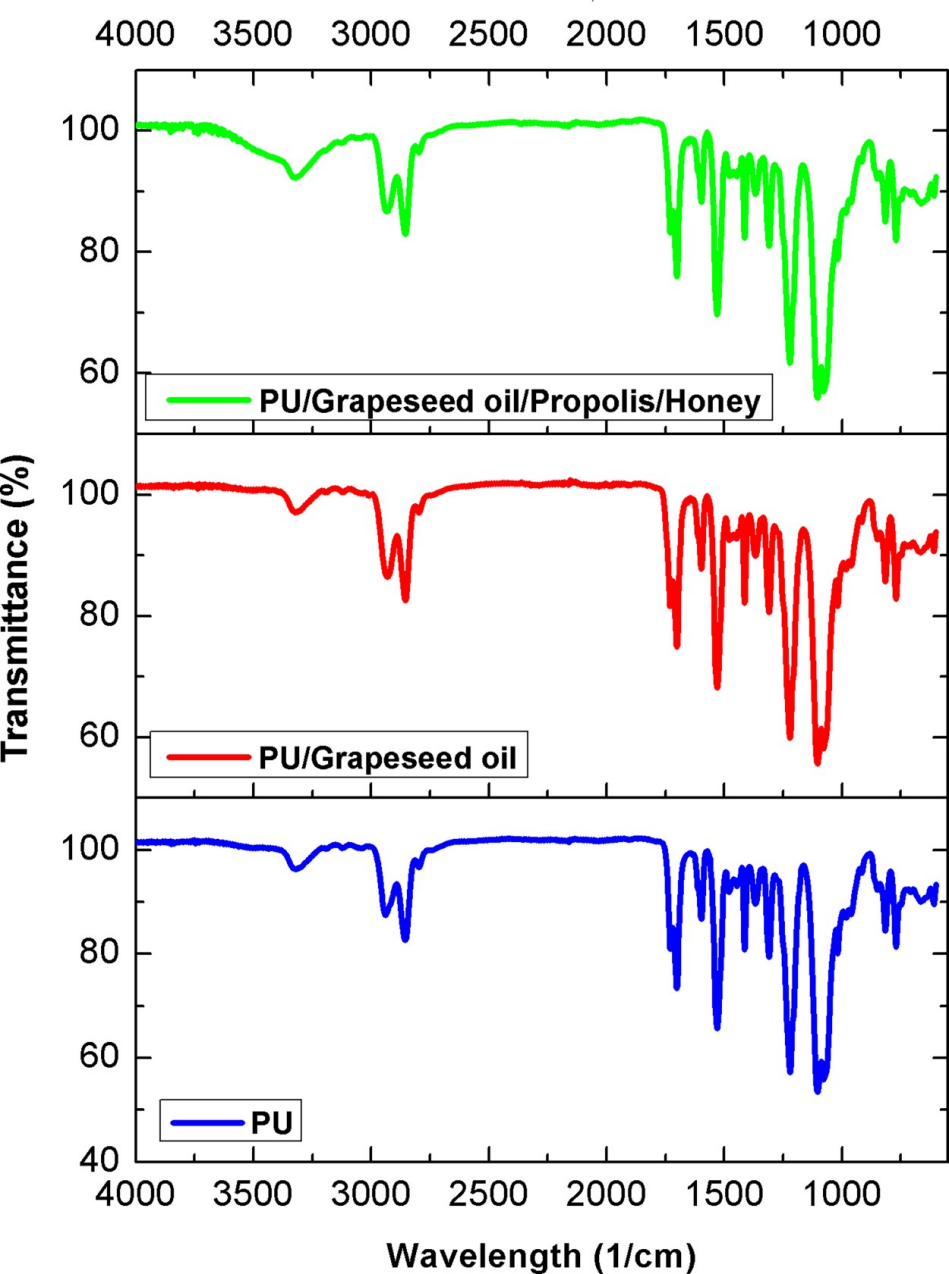
FTIR spectra of a) Polyurethane b) Polyurethane/grape seed oil composites c) Polyurethane/grape seed oil/propolis/honey composites.

Fig 4 indicates the absorption bands present in the electrospun PU, PU/grape seed oil and PU/grape seed oil/honey/propolis scaffold. The spectrum of PU showed a wide band at 3323 cm^−1^ indicating presence of N-H stretching and the peaks at 1597 cm^-1^ and 1531 cm^-1^ denotes the vibration of NH. The peaks seen at 2939 cm^-1^ and 2854 cm^-1^ corresponds to the CH_2_ stretching and the peak at 1413 cm^-1^ was attributed to the vibrations of CH_2_. The C = O stretching corresponding to carboxylic groups was shown by a twin peak at 1730 cm^-1^ and 1703 cm^-1^ and the C-O stretching attributed to alcohol groups were seen at peaks 1221 cm^-1^,1104 cm^-1^ and 1078 cm^-1^ respectively [35]. There were no additional peaks formed in PU/grape seed oil and PU/grape seed oil/honey/propolis scaffold, but there was a change in peak intensity with the incorporation of additives.

### Contact angle measurements

The wettability of electrospun PU, PU/grape seed oil and PU/grape seed oil/honey/propolis scaffold were highlighted. The contact angle of PU was observed to be 100° ± 0.5774 whereas for fabricated PU/grape seed oil/honey/propolis scaffold, the contact angle was found to be 113° ±1.155 and 60° ± 2.082 respectively.

### Thermal stability

**Fig 5:**
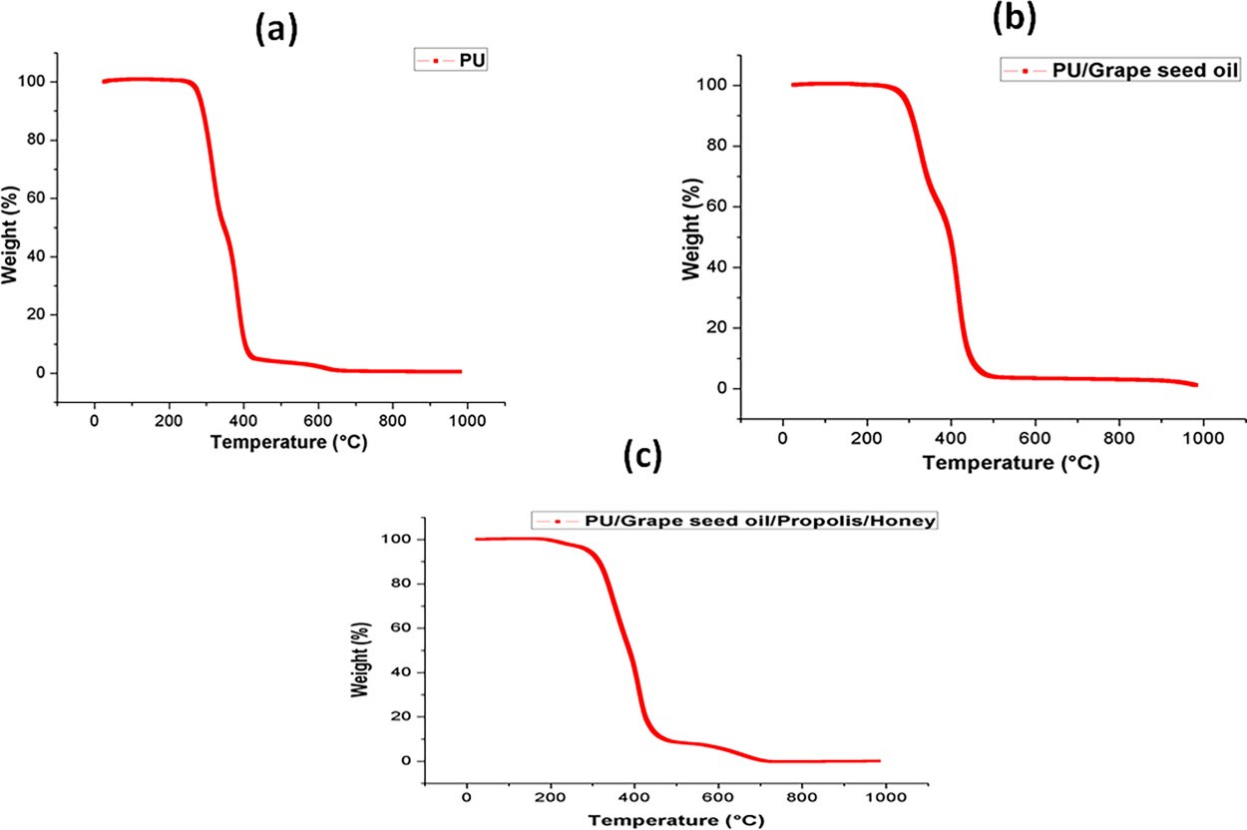
TGA analysis of a) Polyurethane b) Polyurethane/grape seed oil composites c) Polyurethane/grape seed oil/propolis/honey composites.

**Fig 6:**
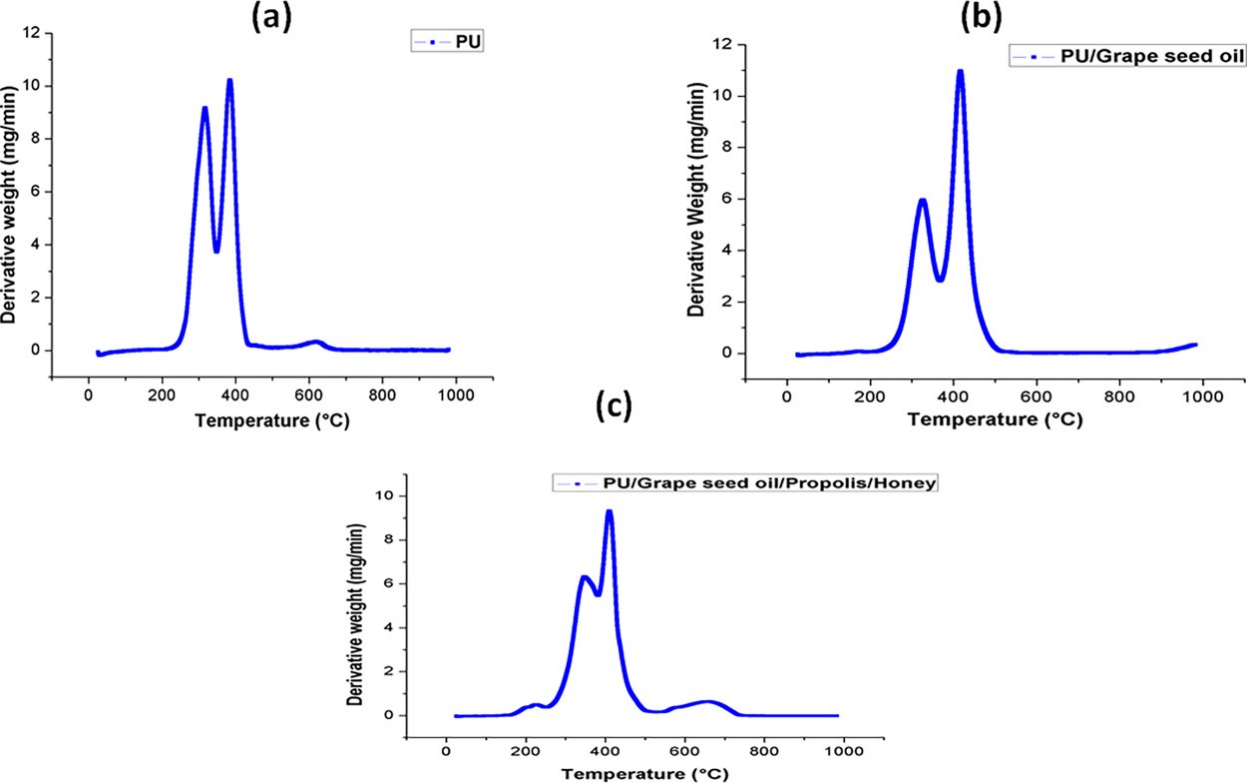
Weight residue percentage of a) Polyurethane b) Polyurethane/grape seed oil composites c) Polyurethane/grape seed oil/propolis/honey composites.

The TGA analysis of electrospun PU, PU/grape seed oil and PU/grape seed oil/honey/propolis scaffold were shown in Fig 5. The initial onset temperature of PU was found to be 276°C whereas, the onset temperatures for electrospun PU/grape seed oil and PU/grape seed oil/propolis/honey scaffold were found to 292°C and 311°C respectively. Further, DTG curve for the electrospun PU/grape seed oil and PU/grape seed oil/propolis/honey scaffold were indicated in Fig 6. The weight loss occurred in the fabricated membranes were indicated in the Table 4.

**Table 4:** Weight loss curves of PU, PU/Grapeseed oil and PU/Grapeseed oil/Propolis/Honey composites obtained from DTG curve.

S.No	First weight loss	Second weight loss	Third weight loss	Fourth weight loss
**PU**	223°C to 348°C	348°C to 446°C	557°C to 684°C	-
**PU/Grapeseed oil**	214°C to 367°C	367°C to 542°C	-	-
**PU/Grapeseed oil/Propolis/Honey**	161°C to 248°C	248°C to 381°C	381°C to 544°C	544°C to 747°C

### Mechanical testing

**Fig 7:**
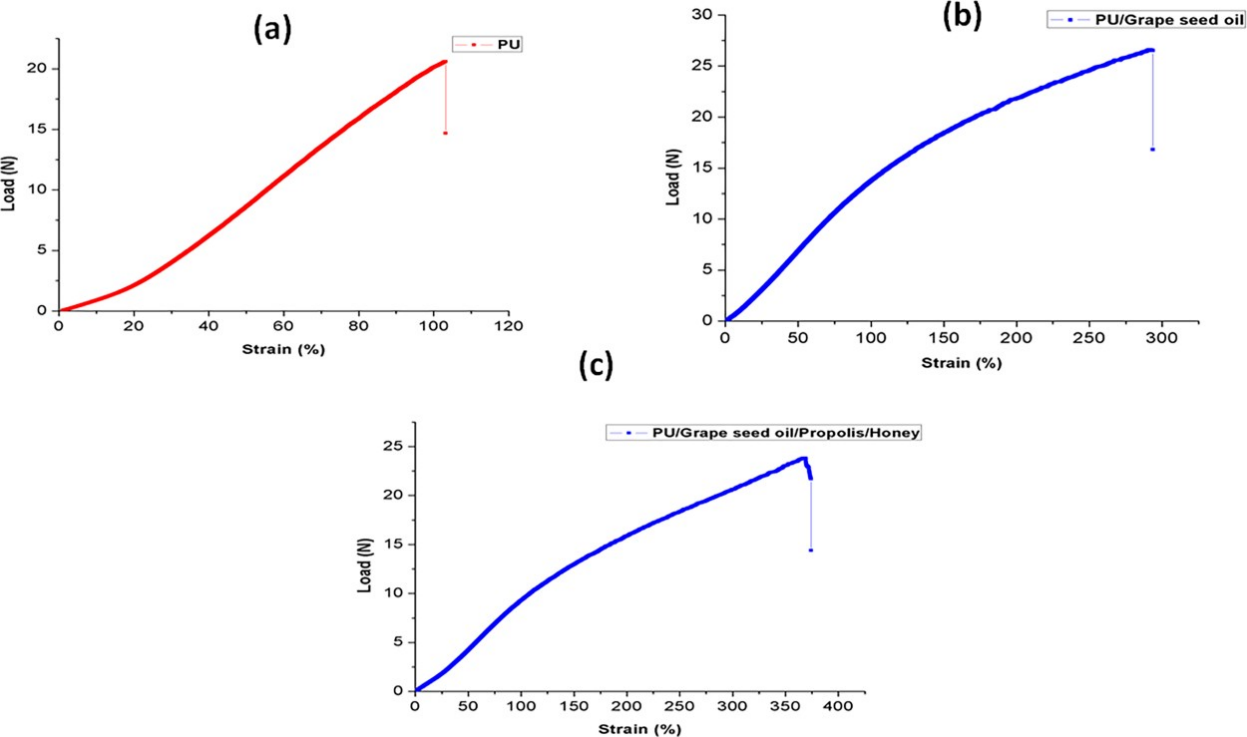
Mechanical testing of a) Polyurethane b) Polyurethane/grape seed oil composites c) Polyurethane/grape seed oil/propolis/honey composite.

The results of mechanical properties for electrospun PU, PU/grape seed oil and PU/grape seed oil/propolis/honey scaffold and shown in Fig 7. The pure PU exhibited tensile strength of 7.12 MPa while for the electrospun PU/grape seed oil and PU/grape seed oil/propolis/honey scaffold, the tensile strength was 12.22 MPa and 16.55 MPa respectively.

### Surface roughness analysis

**Fig 8:**
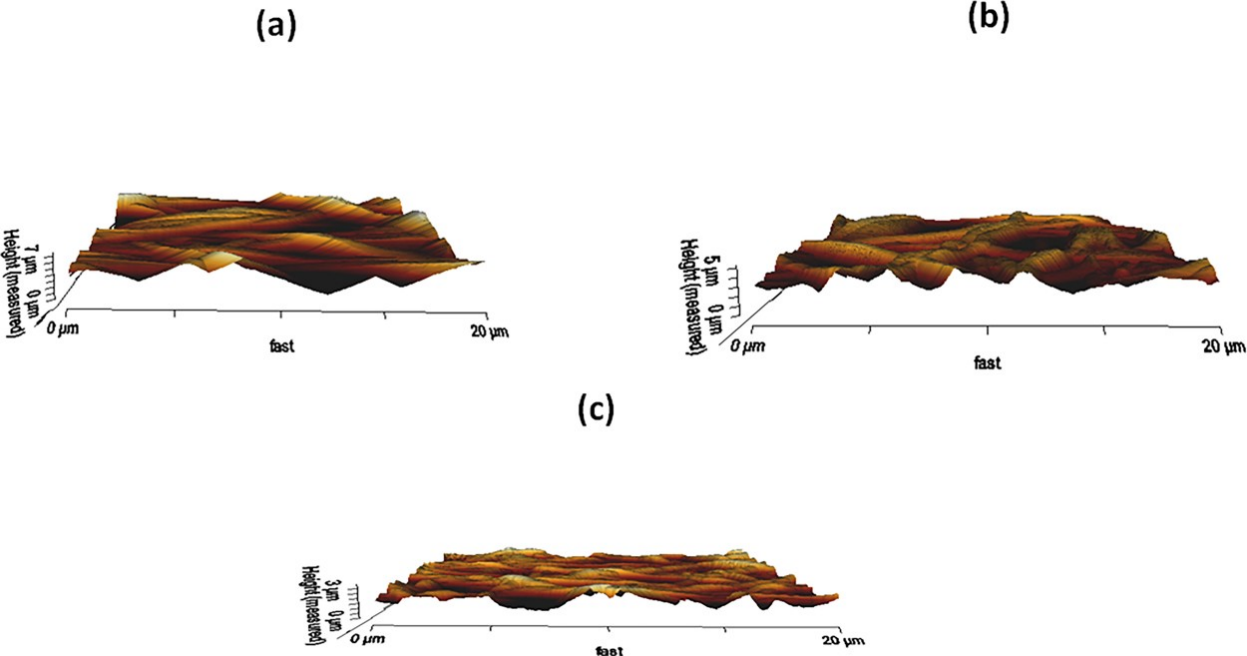
AFM images of a) Polyurethane b) Polyurethane/grape seed oil composites c) Polyurethane/grape seed oil/propolis composites.

The measured surface roughness for electrospun PU, PU/grape seed oil and PU/grape seed oil/propolis/honey scaffold were shown in Fig 8. The measured surface roughness for pristine PU was 576 nm and for the developed PU/grape seed oil and PU/grape seed oil/propolis/honey scaffold, it was found to be 525 nm and 482 nm respectively.

### Blood compatibility analysis

**Fig 9:**
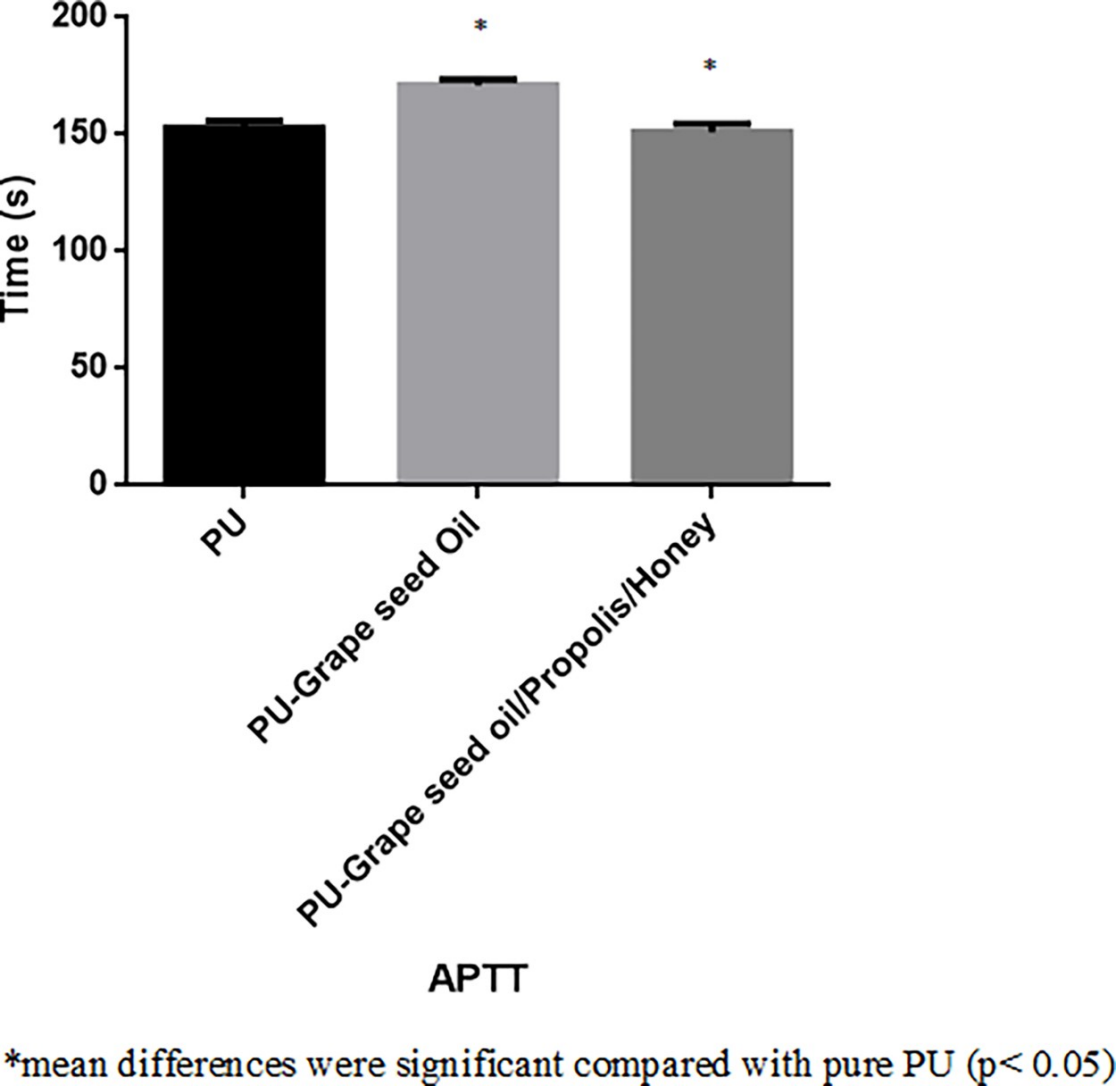
APTT assay of a) Polyurethane b) Polyurethane/grape seed oil composites c) Polyurethane/grape seed oil/propolis/honey composites.

**Fig 10:**
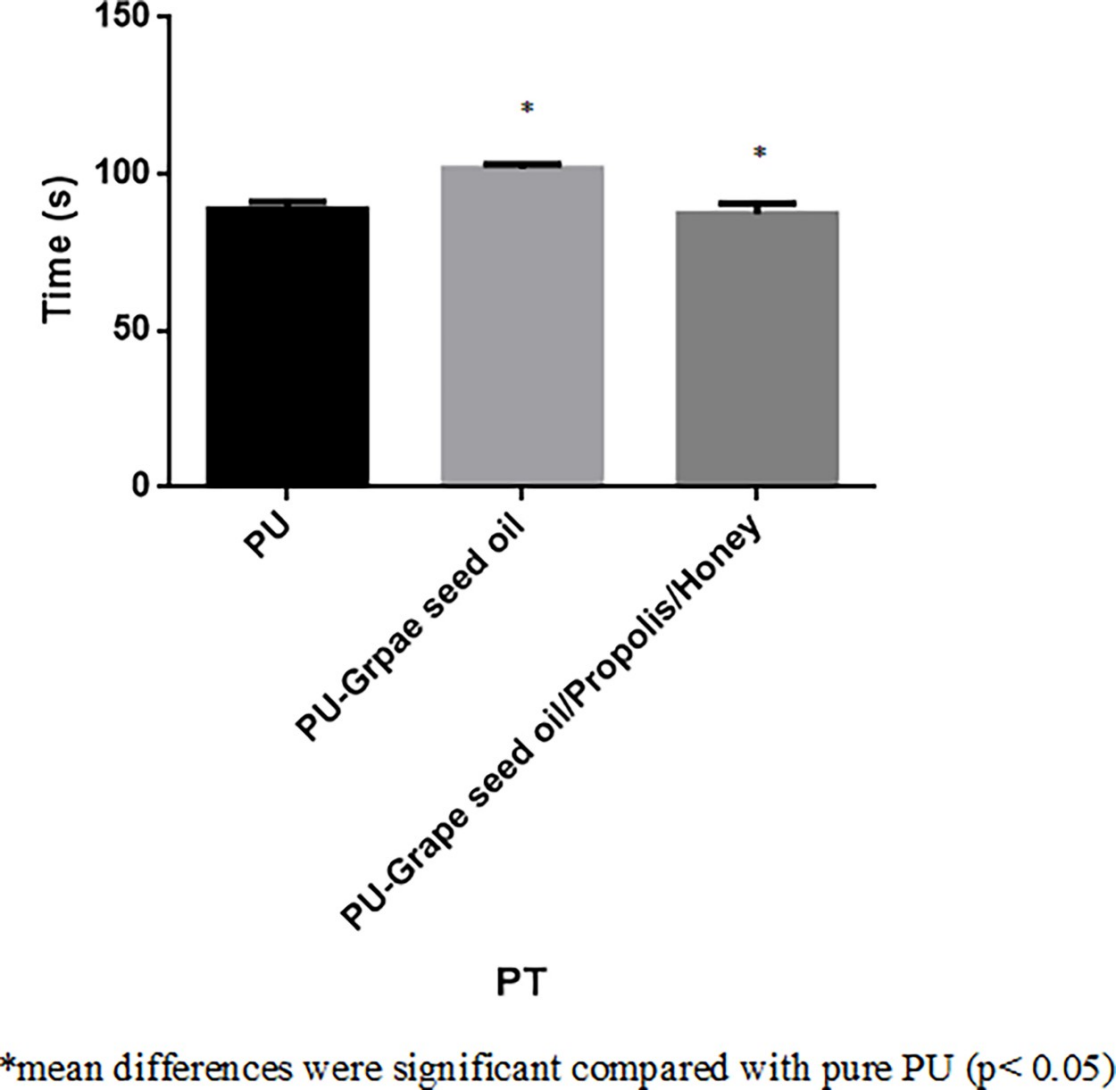
PT assay of a) Polyurethane b) Polyurethane/grape seed oil composites c) Polyurethane/grape seed oil/propolis/honey composites.

**Fig 11:**
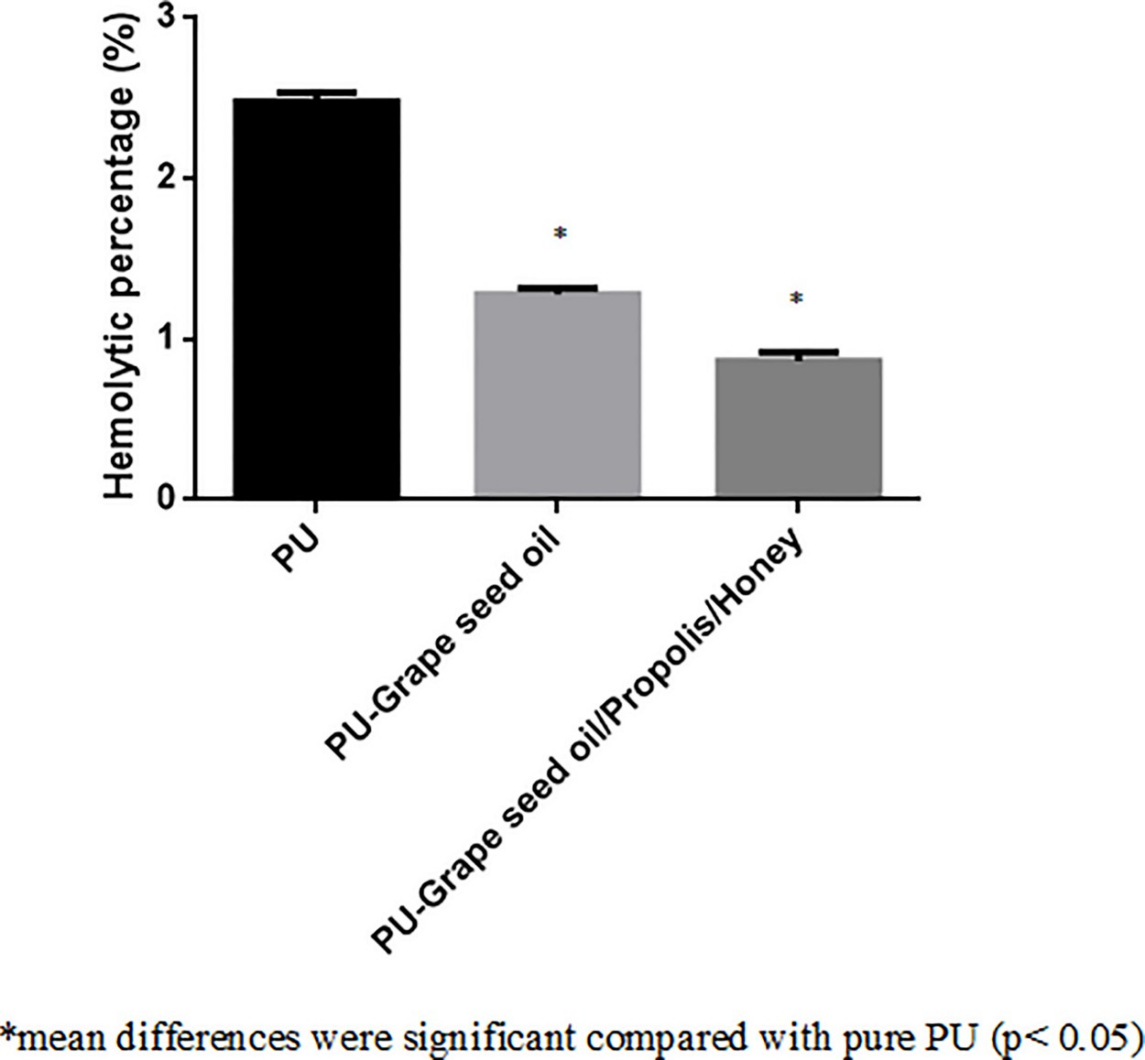
Hemolytic assay of a) Polyurethane b) Polyurethane/grape seed oil composites c) Polyurethane/grape seed oil/propolis/honey composites.

The coagulation assay results for electrospun PU, PU/grape seed oil and PU/grape seed oil/propolis/honey scaffold were shown in Figs 9–11. The APTT for the electrospun PU/grape seed oil and PU/grape seed oil/propolis/honey scaffold was found to be 171 ± 2.646 s and 151 ± 3.606 s, whereas for pristine PU, the APTT was found to be 152.7 ± 3.055 s respectively. Similarly, PT for electrospun PU/grape seed oil and PU/grape seed oil/propolis/honey scaffold were found to be 101.7 ± 1.528 s and 87.33 ± 3.215 s, whereas for pristine PU, the PT was found to be 88.67 ± 2.517 s respectively. Further, for PU, the hemolytic index was found to be 2.48%, while for electrospun PU/grape seed oil and PU/grape seed oil/propolis/honey scaffold, the hemolytic index was observed to be 1.280% and 0.863%.

### Cytocompatibility analysis

**Fig 12:**
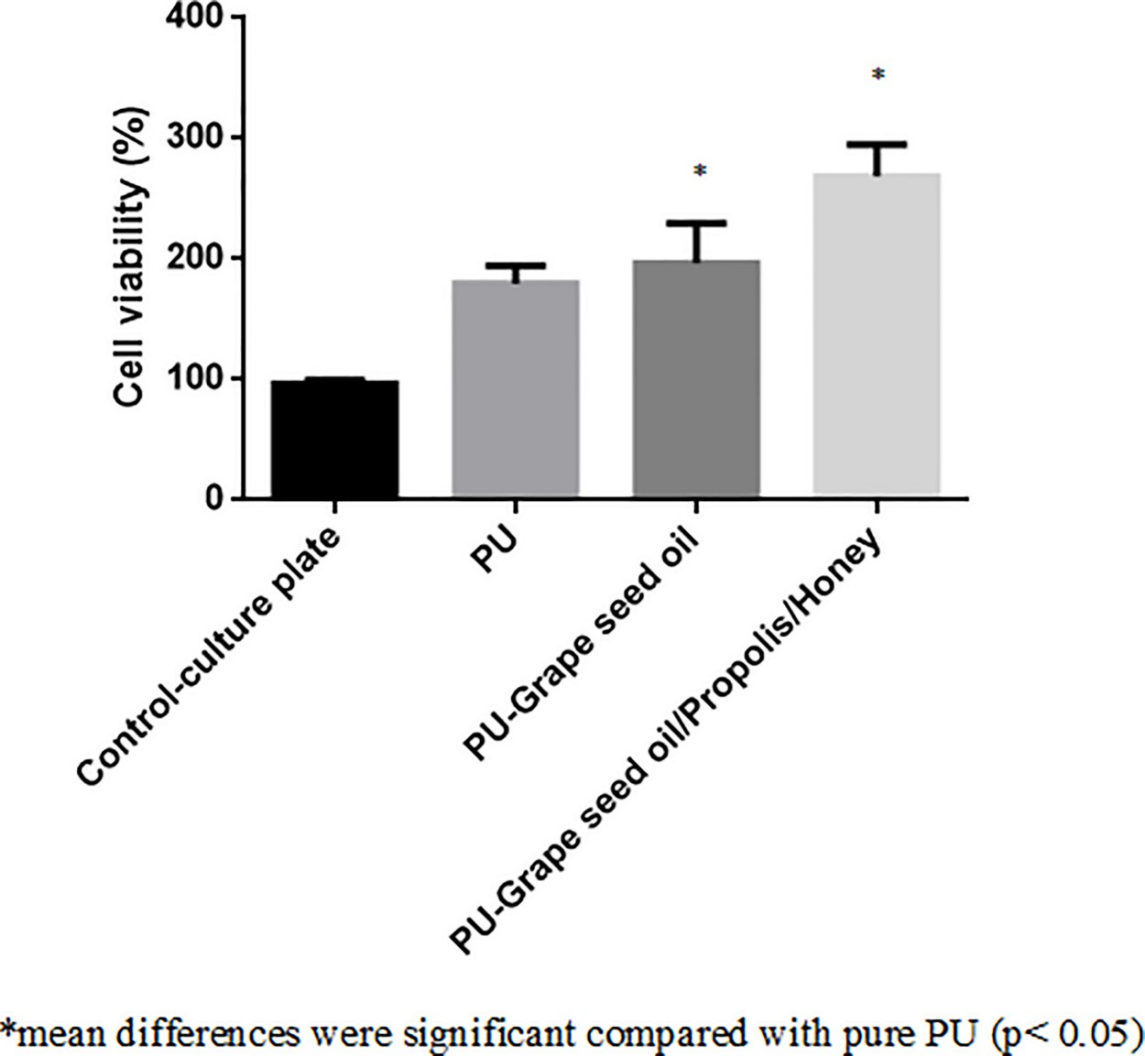
MTS assay of a) Polyurethane b) Polyurethane/grape seed oil composites c) Polyurethane/grape seed oil/propolis/honey composites.

The cell viability of the electrospun nanofibrous PU, PU/grape seed oil and PU/grape seed oil/propolis/honey scaffold using MTS assay were shown in Fig 12. After 3 days culture, the PU membrane showed cell viability of 179.7 ± 15.04% and the electrospun PU/grape seed oil and PU/grape seed oil/propolis/honey scaffold showed viability of 196.7 ± 33.25% and 268.7 ± 26.50% respectively.

## Discussion

The one of the objective in bone tissue engineering is to fabricate and design a scaffold which should mimic the native structure of the bone. Now-a-days, the widely used materials in tissue engineering applications is the electrospun synthetic polymers owing to the excellent resembling of the ECM structure. But, the synthetic materials possess lack in bioactivity which limited their usage in the biomedical applications. The highlight of the research is improving the bioactivity and of the PU through adding grape seed oil, propolis and honey. The grape seed oil, honey and propolis were utilized in this research because of its non-toxic behavior and its medicinal properties. Hence, the polyurethane added with grape seed oil, honey and propolis was electrospun and utilized for bone tissue engineering. To analyze its effect for bone tissue engineering, the various physiochemical characteristics and biocompatibility studies were performed and their results were summarized above. From the SEM investigation, it was observed that the prepared scaffold showed reduction in fiber diameter compared to the pure PU. With the addition of honey and propolis in to the PU matrix, there was a synergistic reduction in the fiber diameter compared to the PU/grape seed oil membrane. Linh et al utilized polyvinyl alcohol for developing bone scaffold incorporated with the gelatin. It was observed that the addition of gelatin into the PVA matrix reduced the fiber diameter and correlates with our findings. Further, the prepared PVA/gelatin scaffold with reduced fiber diameter were observed to be proliferate more number of osteoblast cells compared to the pure PVA [36]. Hence, reduced fiber diameters of developed scaffold found to be appropriate for bone tissue engineering. In IR analysis, it was observed the peak intensity was decreased in PU/grape seed oil scaffold and increased in PU/grape seed oil/honey/propolis scaffold. Unnithan et al electrospun PU scaffold added with emu oil nanofibers. It was observed that the intensity of the PU peak was altered with the addition of emu oil and concluded the reason was owing to the formation of hydrogen bond. In our study, the change in the intensity can be attributed to the hydrogen bond formation [37]. Further, it have been reported that the formation of hydrogen bonds during the combination of two different macromolecules will be stronger compared to the bonding between the molecules of the same polymer [38]. In our electrospun PU/grapeseed oil and PU/grapeseed oil/honey/propolis, the stronger inter-hydrogen bonds was formed because of interaction of NH of PU and CH/OH present in the grapeseed oil, honey and propolis. Moreover, there was slight shift of CH band was seen at 2939 cm^-1^ in PU to 2931 cm^-1^ and 2933 cm^-1^ in PU/grape seed oil and PU/grape seed oil/honey/propolis scaffold. Jaganathan et al fabricated polyurethane membrane added with corn and neem oil. It was showed that the addition of corn and neem oil showed peak shifts concluding the interaction of PU with corn and neem oil. In our study, the fabricated nanocomposites showed CH peak shifts which confirms the presence of additives in the polyurethane matrix [39]. The wettability analysis depicted that the electrospun PU/grape seed oil scaffold showed hydrophobic nature while the PU/grape seed oil/honey/propolis scaffold exhibited hydrophilic nature. Designing scaffolds with optimum wettability is a criteria for enhanced cell adhesion of osteoblasts. Recent research suggested scaffolds with the contact angle below 106° is found to facilitate the osteoblast cell adhesion and proliferation [40]. In our case, blending of polyurethane with grapeseed oil resulted in the contact angle above this margin. Hence, in this work an effort to impart the wettability to the PU/grape seed oil by blending with honey/propolis. A recent study favored our assumption where they depicted the addition of honey and propolis rendered the scaffolds to be hydrophilic [24, 41]. Further, the constituents present in the honey and propolis may be promoting the bioactivity of the scaffold. To our expectation, addition of honey and propolis resulted in the contact angle reduction and rendered the surface hydrophilic which may be conducive for bone tissue ingrowth. Abdal Hay et al. utilized nylon-6 for developing the bone scaffold incorporated with hydroxyapatite. It was reported that the developed scaffold with hydrophilic nature resulted in the significant formation of apatite layers compared to control [42]. Hence, our engineered electrospun scaffolds with the increased wettability may invigorate the formation of apatite layers for new bone formation. In thermal analysis, it was observed that the electrospun PU/grape seed oil and PU/grape seed oil/honey/propolis scaffold exhibited higher thermal stability because of addition of grape seed oil, honey and propolis compared to the pristine PU. Jaganathan et al prepared scaffold based on PU scaffold incorporated with mustard oil nanofibers. It was reported that the incorporation of mustard oil into the PU membrane improved the thermal stability and resembles our findings [43]. Further, in the DTG analysis, the first weight loss peak intensity in the electrospun PU/grape seed oil scaffolds was decreased than the PU indicating their reduced weight loss, while that weight loss peak intensity was slightly increased than the PU/grape seed oil membrane when adding honey and propolis in the PU matrix. However, the weight loss peak intensity of the PU was observed to be decreased in both fabricated nanocomposites indicating its reduced weight loss. Similar to the thermal stability improvement, the addition of grape seed oil, honey and propolis enhanced the tensile strength of the PU. Salifu et al prepared scaffold for bone tissue engineering based on gelatin blended with hydroxyapatite. It was observed that the gelatin/ hydroxyapatite showed tensile strength in the range of 4 to 10 MPa and concluded it as a suitable candidate for the bone tissue engineering [44]. Our tensile results of developed scaffold were observed to be better than the reported values indicating its superiority in bone tissue engineering. In surface roughness measurements, the electrospun nanocomposites showed reduced the surface roughness compared to pure PU. Hence, they exhibit smooth surfaces than the pristine PU. It was reported that the osteoblast cells prefer smooth surfaces for enhanced adhesion and proliferation [45]. Hence, the surface of the developed PU/grape seed oil and PU/grape seed oil/propolis/honey scaffold might be conducive for the enhanced osteoblast adhesion and proliferation. In blood compatibility measurements, it was found that the electrospun PU/grapeseed oil were found to be blood compatible by prolonging the coagulation times compared to the pure polyurethane. Since, the surface of electrospun PU/grape seed oil was found to be hydrophobic which allows the adhesion of plasma proteins irreversibly resulting in the enhancement of blood compatibility. However, the addition of honey and propolis to the PU/grapeseed oil there is a slight reduction of blood clotting time but it was similar range to pure PU. This behavior may be attributed to the balance of the polar and apolar constituents present in the honey/propolis [46]. Further, in the hemolytic assay measurements, both electrospun nanocomposites showed lesser hemolytic index than the pristine PU indicating their less toxicity to the red blood cells. Since, the hemolytic index of the developed scaffold was below 1% and it was considered as non-hemolytic material [41]. Finally, the cell viability of the both electrospun nanocomposites were found to be enhanced compared to the pristine PU owing to presence of olive oil, honey and propolis. Moreover, the hydrophilic PU/grapeseed/honey/propolis scaffolds rendered enhanced cell viability compared to PU/grapeseed oil. This enhanced cell adhesion of fibroblast may be due to the contact angle of this scaffold (60°) lies within the optimal range of maximum cell proliferation as reported recently [47].

## Conclusion

In this work, we presented a fabrication and testing new novel scaffold based on PU added with grape seed oil, honey and propolis using electrospinning technique The diameter of the electrospun PU/grape seed oil and PU/grape seed oil/honey/propolis scaffold were observed to be reduced compared to pristine PU control. The existence of grape seed oil, honey and propolis in PU was identified by CH band peak shift and also hydrogen bond formation. The contact angle of PU/grape seed oil scaffold was found to increase owing to hydrophobic nature and the contact angle for the PU/grape seed oil/honey/propolis were decreased because of hydrophilic nature. Further, the prepared PU/grape seed oil and PU/grape seed oil/honey/propolis scaffold showed enhanced thermal stability and reduction in surface roughness than the control revealed in TGA and AFM analysis. Moreover, the developed scaffold displayed delayed blood clotting time than the PU control proved enhanced blood compatibility. The hemolytic assay and cytocompatibility studies revealed that the electrospun PU/grape seed oil and PU/grape seed oil/honey/propolis scaffold possess non-toxic to RBC and HDF cells indicating better blood compatibility and cell viability rates. Hence, the present study concludes that the newly electrospun nanofibrous composite scaffold with desirable characteristics that might be used as alternative candidate for bone tissue engineering. However, it would be interesting to examine the toxicity using specific test like live dead assay which may provide further light on the cytocompatibility behavior.
